# The role of NPY2R/NFATc1/DYRK1A regulatory axis in sebaceous glands for sebum synthesis

**DOI:** 10.1186/s11658-023-00467-4

**Published:** 2023-07-27

**Authors:** Tao Yang, Renyi Hei, Xiaosong Li, Tianhua Ma, Yifen Shen, Chao Liu, Wen He, Lin Zhu, Yongchun Gu, Yanping Hu, Wenbin Wei, Yihang Shen

**Affiliations:** 1 Department of Medical Cosmetology, Suzhou Ninth People’s Hospital, Suzhou, 215200 Jiangsu China; 2 Department of Otolaryngology-Head and Neck Surgery, General Hospital of Shenyang Military Area Command, Shenyang, 110016 Liaoning China; 3 Department of Anorectal Surgery, Suzhou Ninth People’s Hospital, Suzhou, 215200 Jiangsu China; 4 Graduate School of Soochow University, Suzhou, 215031 Jiangsu China; 5 Central Laboratory, Suzhou Ninth People’s Hospital, 2666, Ludang Road, Suzhou, 215200 Jiangsu China; 6 Department of Molecular Pathology, The Affiliated Cancer Hospital of Zhengzhou University, Henan Cancer Hospital, Zhengzhou, 450008 Henan China; 7 Department of Oral Surgery, Shanghai Ninth People’s Hospital, Shanghai Jiao Tong University School of Medicine, College of Stomatology, Shanghai Jiao Tong University; National Center for Stomatology; National Clinical Research Center for Oral Diseases; Shanghai Key Laboratory of Stomatology, No. 639 Zhizaoju Road, Huangpu District, Shanghai, 200013 China

**Keywords:** Sebaceous glands, NPY2R, NPY5R, NFATc1, DYRK1A, Puberty onset

## Abstract

**Background:**

Sebaceous glands (SGs) synthesize and secret sebum to protect and moisturize the dermal system via the complicated endocrine modulation. Dysfunction of SG are usually implicated in a number of dermal and inflammatory diseases. However, the molecular mechanism behind the differentiation, development and proliferation of SGs is far away to fully understand.

**Methods:**

Herein, the rat volar and mammary tissues with abundant SGs from female SD rats with (post-natal day (PND)-35) and without puberty onset (PND-25) were arrested, and conducted RNA sequencing. The protein complex of Neuropeptide Y receptor Y2 (NPY2R)/NPY5R/Nuclear factor of activated T cells 1 (NFATc1) was performed by immunoprecipitation, mass spectrum and gel filtration. Genome-wide occupancy of NFATc1 was measured by chromatin immunoprecipitation sequencing. Target proteins’ expression and localization was detected by western blot and immunofluorescence.

**Results:**

NPY2R gene was significantly up-regulated in volar and mammary SGs of PND-25. A special protein complex of NPY2R/NPY5R/NFATc1 in PND-25. NFATc1 was dephosphorylated and activated, then localized into nucleus to exert as a transcription factor in volar SGs of PND-35. NFATc1 was especially binding at enhancer regions to facilitate the distal SG and sebum related genes’ transcription. Dual specificity tyrosine phosphorylation regulated kinase 1A (DYRK1A) contributed to NFATc1 phosphorylation in PND-25, and inactivated of DYRK1A resulted in NFATc1 dephosphorylation and nuclear localization in PND-35.

**Conclusions:**

Our findings unmask the new role of NPY2R/NFATc1/DYRK1A in pubertal SG, and are of benefit to advanced understanding the molecular mechanism of SGs’ function after puberty, and provide some theoretical basis for the treatment of acne vulgaris from the perspective of hormone regulation.

**Graphical Abstract:**

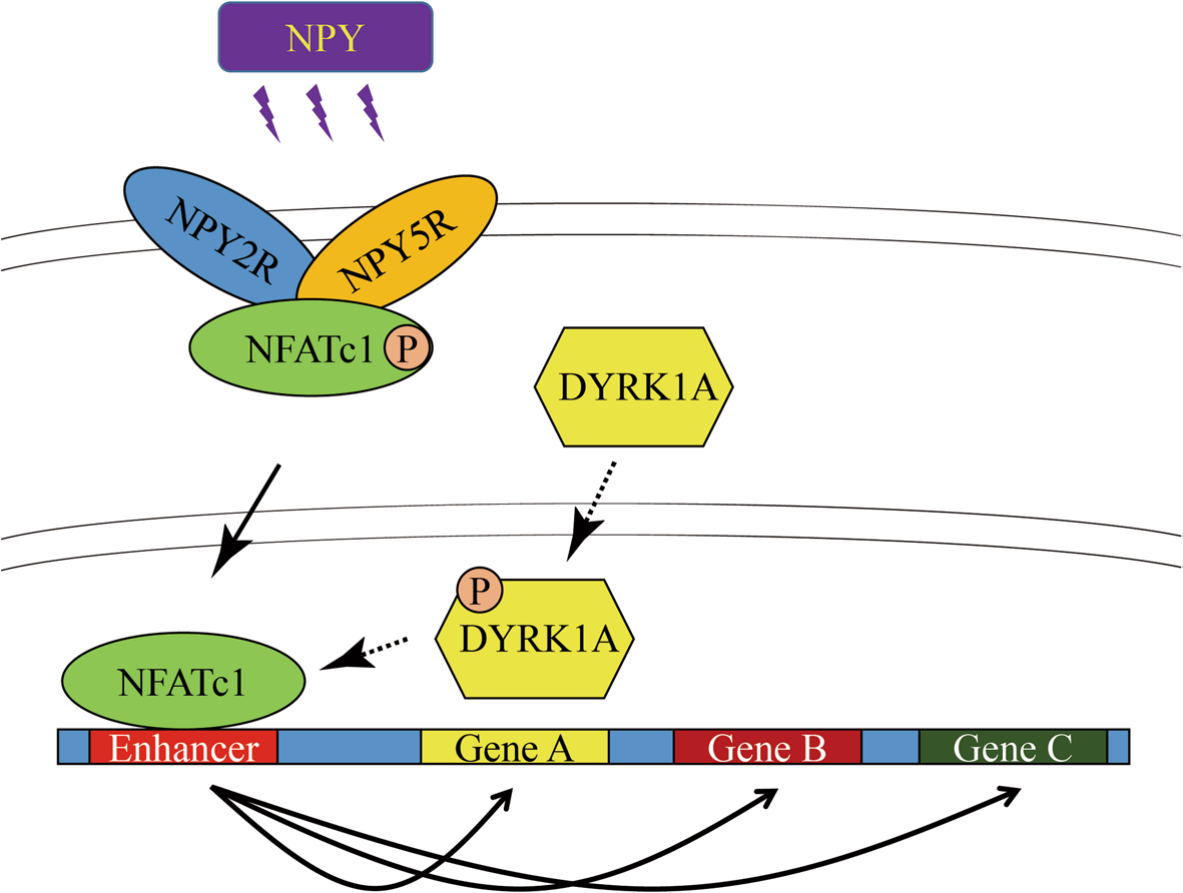

**Supplementary Information:**

The online version contains supplementary material available at 10.1186/s11658-023-00467-4.

## Background

As the largest organ, skin serves as the body’s natural protective barrier, which is composed of the epidermal cells as well as the epidermal surface lipid film and natural moisturizing factors that populate them. Epidermal surface lipid film is a kind of transparent lipid “membrane” with weak acidity mixed by the emulsification of sebum and sweat [1]. Sebum is synthesized by sebaceous glands (SGs), epidermal keratinocytes and sweat glands via a unique holocrine manner, in which sebocytes undergo the differentiation, maturation, transformation into sebum vacuoles, programmed death and disintegration followed by the release of intracellular substances [2]. Different from lipid substances (triglycerides, free fatty acid and cholesterol) derived from the other sources, sebum produced by SGs contains special squalene, wax ester, galactose, vitamins and some antimicrobial peptides, but extremely low cholesterol. Sebum also serves as a barrier to retard the release of skin odour signals produced by sweat glands and SGs [3, 4]. In a nutshell, sebum plays a crucial role in protecting and moisturizing the dermal system.

Due to the major distribution in scalp and central face (400–900 glands/cm^2^) compared to the average density 100/cm^2^ throughout the entire skin system [5, 6], dysfunction of SGs and irregular sebum secretion are usually implicated in chronic inflammatory diseases, including particular facial acne, but also atopic dermatitis, seborrhea, psoriasis and rosacea. Previous observations in clinical and experimental dermatology have highlighted the importance of nervous and neuroendocrine system majorly three neuroendocrine axes including hypothalamic-pituitary-thyroid (HPT) axis, hypothalamic-pituitary-adrenocortical (HPA) axis and hypothalamic-pituitary-gonad (HPG) axis that are implicated in modulation of cutaneous physiology and pathology. Numerous hormones, peptides and transmitters from pituitary and hypothalamus communicate with skin and its appendages through a large number of receptors [7, 8]. Beyond that, various neuropeptides and sterol hormones and cytokines from peripheral nerve fiber, capillaries, lymphatic capillaries and immune cells are also involved in the epidermal microenvironment as well as affect the proliferation and differentiation of sebocytes in burn or wound healing and tissue regeneration [9–11]. Nevertheless, we still have a long way to understand the molecular mechanism behind the differentiation, development and proliferation of sebaceous glands systematically and in depth.

We are more concerned about the abnormal function of SGs in adolescent acne. During pubertal onset, a number of hormones and neuropeptides are changing [12, 13]. In addition to the known distal modulations of androgen, growth factor, prolactin, adrenocorticotropin and melanotropin [8], we hope to seek more novel proximal regulators unmasking the substantial effect on SGs. In present research, we isolated the sebaceous gland tissue from rats with (post-natal day (PND)-35) and without puberty onset (PND-25) as previously described [14, 15] and conducted transcriptomic sequencing, and focused on neuropeptide Y receptor Y2 (*NPY2R*) gene. Then we further studied the subsequent intracellular and endonuclear signaling pathway for final gene expression regulation in SGs. Our findings are of benefit to advanced understanding the molecular mechanism of SGs’ function after puberty, and provide some theoretical basis for the treatment of acne vulgaris from the perspective of hormone regulation.

## Methods

### Animal study

Total twenty female Sprague Dawley (SD) rats with post-natal day (PND)-25 (*n* = 10) and PND-35 (*n* = 10) were housed at 25 °C with enough food and water. Ten mg/kg NPY2R antagonist SF-11 (sc-311535, Santa Cruz Biotechnology) [16] was subcutaneously administrated to PND-35 (*n* = 5) in designated position for 24 h. One mg/kg DYRK1-IN-1 (HY-132308, MedChemExpress) [17] was subcutaneously administrated with PND-25 (*n* = 5) in designated position for 24 h. After sacrificing by cervical dislocation, skin tissues of volar, nipple and abdominal region were disinfected with alcohol (abdominal needs to be removed before disinfection). The whole skin was separated from the underlying tissue, and cut into 1 cm^2^, and stored in − 80 °C for sequencing and Oil red O staining, or in 4% paraformaldehyde for morphological detection. Three rats were used for all morphological experiments such as H&E, Oil red O, immunofluorescence assay*.* Two rats were used for molecular study including western blot, gel filtration, immunoprecipitation and sequencing. All animal experiments were performed in accordance with the Declaration of Helsinki and “Guide for The Care and Use of Laboratory Animals” 8^th^ Edition, and approved by the Committee on the Ethics of Suzhou Ninth People’s Hospital (Assigned number: SZJY-2022-08).

### H&E staining

Tissues were processed paraffin embedded, and sectioned 4 μm on a microtome. The sections were dewaxed through xylene—gradient alcohol, and stained using Hematoxylin and Eosin Kit (Beyotime Biotechnology). After that, sections were dehydrated through gradient alcohol–xylene.

### Oil red O staining

Frozen skin tissues were embedded by OCT (SAKURA), and sectioned 4 μm directly on a freezing microtome. Sections were washed by 50% alcohol and added improved oil Red O dye solution (Beyotime Biotechnology) to cover the whole tissue for 20 min, then washed by washing buffer for 30 s and PBS for 20 s. After that, sections were further processed by Hematoxylin and Eosin Kit without dehydration.

### Immunofluorescence

The steps prior to dewaxing were consistent with H&E staining. Sections were processed by 3% hydrogen peroxide for 3 min, and rinsed with running water. After 1 min antigen repair using Tris–EDTA Antigen Repair Solution (PR30002, Proteintech), sections were washed by PBS for three times, and blocked by 5% BSA in room temperature for 30 min, then the primary antibodies including KRT5 (1: 200, #66727-1-lg, Proteintech), KRT10 (1: 200, #18343-1-AP, Proteintech), NPY1R (1: 200, ab91262, Abcam), NPY2R (1: 200,SAB4502029, MilliporeSigma), NPY5R (1: 200, ab133757, Abcam), p-NFATc1 (Ser294) (1: 200, AF8012, Affinity Biosciences), NFATc1 (1: 200, #66963-1-lg, Proteintech) or DYRK1A (1: 200, A0595, Abclonal) were added to incubate at 4 °C overnight. Appropriate secondary antibodies including FITC-conjugated affinipure goat anti-mouse IgG (H + L) (1: 10000, SA00003-1, Proteintech), FITC-conjugated affinipure goat anti-rabbit IgG (H + L) (1: 10000, SA00003-2, Proteintech), CoraLite 594-conjugated Donkey anti-mouse IgG (H + L) (1: 20000, SA00013-7, Proteintech) and CoraLite 594-conjugated Donkey anti-rabbit IgG (H + L) (1: 20000, SA00013-8, Proteintech) were incubated at room temperature avoiding light for 1 h. After PBS washing three times and drying in air, sections were added by mounting medium with DAPI-aqueous, fluoroshield (ab104139, Abcam) and covered by cover glass.

### RNA sequencing

The main procedures of this experiment included RNA extraction, library construction, quality control, sequencing and analysis as described in our previous studies [15, 18]. The clean reads were aligned to rat genome assembly Rnor_6.0.

### Western blot

General western blotting was described previously [19, 20]. The information, concentration and treatment condition of antibodies were listed as follows (antibodies were sorted according to the order in which they appeared), NPY1R (1: 2000, ab91262, Abcam), NPY2R (1: 2000, SAB4502029, MilliporeSigma), NPY5R (1: 2000, ab133757, Abcam), GAPDH (1: 5000, AC001, Abclonal), NFATc1 (1: 2000, #66963-1-lg, Proteintech), p-NFATc1 (Ser294) (1: 1000, AF8012, Affinity Biosciences), CDC2 (1: 2000, #9116, CST), p-CDC2 (Tyr15) (1: 1000, #9111, CST), PIM-1 (1: 2000, #54523, CST), p-PIM-1 (Tyr218) (1: 500, orb6723, Biorbyt), mTORC1 (1: 2000, A2445, Abclonal), p-mTORC1 (Ser2448) (1: 1000, AP0094, Abclonal), IKKε (1: 2000, A16470, Abclonal), p-IKKε (Ser172) (1: 1000, #8766, CST), MAP3K8 (1: 2000, A15623, Abclonal), p-MAP3K8 (Ser400) (1: 1000, #4491, CST), DYRK1A (1: 2000, A0595, Abclonal), p-DYRK1A (Tyr271/319) (1: 1000, AF3507, Affinity Biosciences), HRP-conjugated affinipure goat anti-rabbit IgG (H + L) (1: 5000, SA00001-2, Proteintech) and HRP-conjugated affinipure goat anti-mouse IgG (H + L) (1: 5000, SA00001-1, Proteintech).

### Gel filtration

The gel filtration assay was performed as previously described [21]. In brief, hypotonic buffer with NP-40 was used to separate the cytoplasm and nuclei of tissues containing 1 × 10^8^ cells and the cytoplasmic proteins were isolated using low salt extraction buffer [20 mM HEPES (pH 7.9), 50 mM NaCl, 25% glycerol, 1.5 mM MgCl2, 0.2 mM EDTA, 0.5 mM dithiothreitol, and protease inhibitors]. The cytoplasmic extracts (4 mg) were directly applied to a Sepharose 6B column (MilliporeSigma) equilibrated with column running buffer containing 20 mM HEPES (pH 7.9), 200 mM NaCl, 1 mM dithiothreitol, 0.1 mM phenylmethylsulfonyl fluoride, and 10% glycerol. Fractions of 1 mL each were collected and NPY1R, NPY2R, NPY5R and NFATc1 were detected by western blot.

### Immunoprecipitation

Frozen tissues were pulverized using ceramic grinding rod in liquid nitrogen, and fixed by 1 mL 4% paraformaldehyde for 10 min, then added glycine to 0.2 M for quenching fixation. After 14,000 rpm centrifugation for 10 min as well as supernatant removal, cell precipitation was added 1 mL cold standard RIPA buffer (#20-188, Millipore) and incubated on ice for 30 min. After 14,000 rpm centrifugation again, supernatant was transferred into another new eppendorf tube. Ultrasonication optional for protein Immunoprecipitation, but necessary for chromatin immunoprecipitation was set as high power 5 s, break 30 s for 35 cycles by CHIP-300 T System (Jiangsu Gaozhou Technology). 20 μL was stored as input, and could be subsequently extracted the total protein or genomic DNA. The rest supernatant was incubated with 1 μg appropriated primary antibodies including NPY5R (ab133757, Abcam) or NFATc1 (MA3-024, Thermo Fisher Scientific) at 4 °C overnight. In next morning, 20 μL Magna ChIP Protein A + G Magnetic Beads (#16-663, Millipore) were added and incubated for another 2 h. Beads were washed by modified washing buffer I [RIPA buffer plus 500 mM LiCl for ChIP, plus 350 mM NaCl for protein IP] for three times, then washed by washing buffer II [RIPA buffer plus 150 mM NaCl] for three times, and finally added 20 μL RIPA buffer. For ChIP, another 480 μL RIPA buffer was added, and added 500 μL Tris-phenol for DNA isolation by general phenol chloroform. Library construction, quality control, sequencing and analysis were described in our previous studies [15]. For protein IP, 5 μL 5 × SDS-PAGE Protein Loading Buffer (Sangon Biotech) was added, and run 10% SDS-PAGE gel. Coomassie brilliant blue staining for interested protein band by mass spectrum (OE Biotech), or western blot for verification of protein–protein interaction.

### Statistical analysis

Data are presented as means ± standard deviations. Student’s *t*-test was used to analyze the intergroup differences. The *p* value less than 0.05 was considered as statistical significance.

## Results

### Sebaceous glands became vibrant after puberty onset

**Fig. 1:**
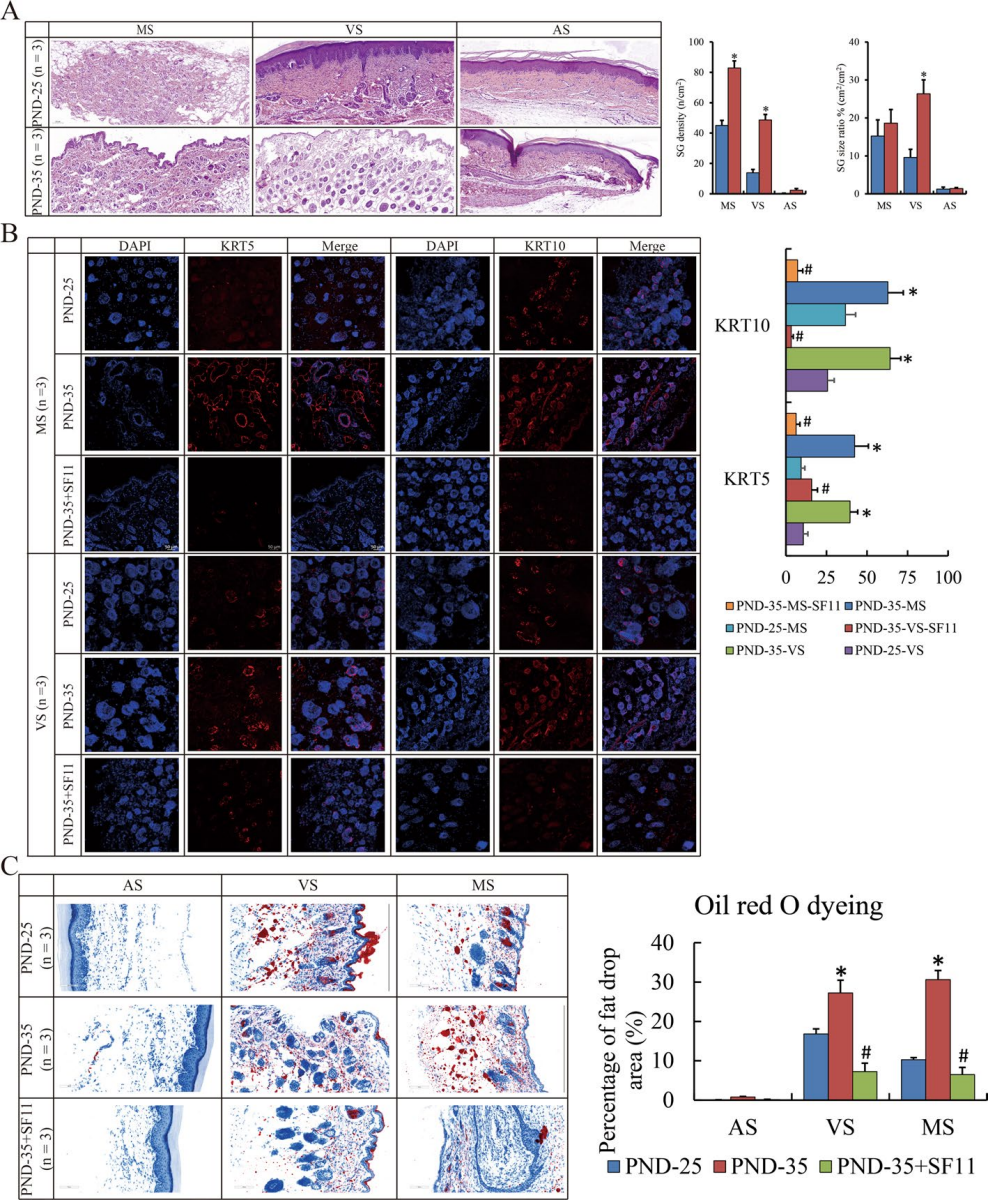
Abundant SG distributed in pubertal skin. **A** H&E staining show the SG count and size in MS, VS and AS of PND-25 and PND-35 rat (magnification ×100). **B** IF show the expression of KRT5 and KRT10 in MS and VS of PND-25, PND-35 and PND-35 with SF-11 (magnification ×200). **C** Oil red O staining show the lipid droplet content in MS, VS and AS of PND-25, PND-35 and PND-35 with SF-11 (magnification ×200). The SG specimens from three individuals were used for immunofluorescence. Ten visual fields were randomly selected to count the positive staining, and calculate the proportion and statistical significance using Student’s *t*-test. “*” and “#” represent *p*-value less than 0.05 compared to the prior group. H&E: Hematoxylin and eosin staining; SG: sebaceous gland; PND: post-natal day; MS: mammary skin; VS: volar skin; AS: abdominal skin; IF: immunofluorescence; IP: immunoprecipitation; SF-11: NPY2R antagonist; DYRK1-IN-1/IN: DYRK1A inhibitor; DEGs: differential expressed genes; NFATc1: nuclear factor of activated T cells 1

Initially, skin tissue of volar, nipple and abdominal regions from juvenile (PND-25) and pubertal (PND-35) female SD rats were obtained. In general, SGs were mainly enriched in volar and mammary skin. We observed that SGs were gigantic and numerous especially in volar skin in PND-35 compared to PND-25 (Fig. 1A). Moreover, robust expression of KRT5 (biomarker of progenitor cells) and KRT10 (biomarker of ductal and sebocytic cells) [22] in PND-35 compared to PND-25 (Fig. 1B). Consistently, Oil red O staining was conducted to investigate the status of sebum synthesis and secretion, and sebum levels in tissues with volar and mammary skin were higher in PND-35 compared to PND-25 (Fig. 1C). The results above determined that differentiation of SGs and sebum synthesis and secretion initiated were substantially activated in pubertal rats.

### NPY2R connected with the suppressive effects on sebaceous glands

**Fig. 2:**
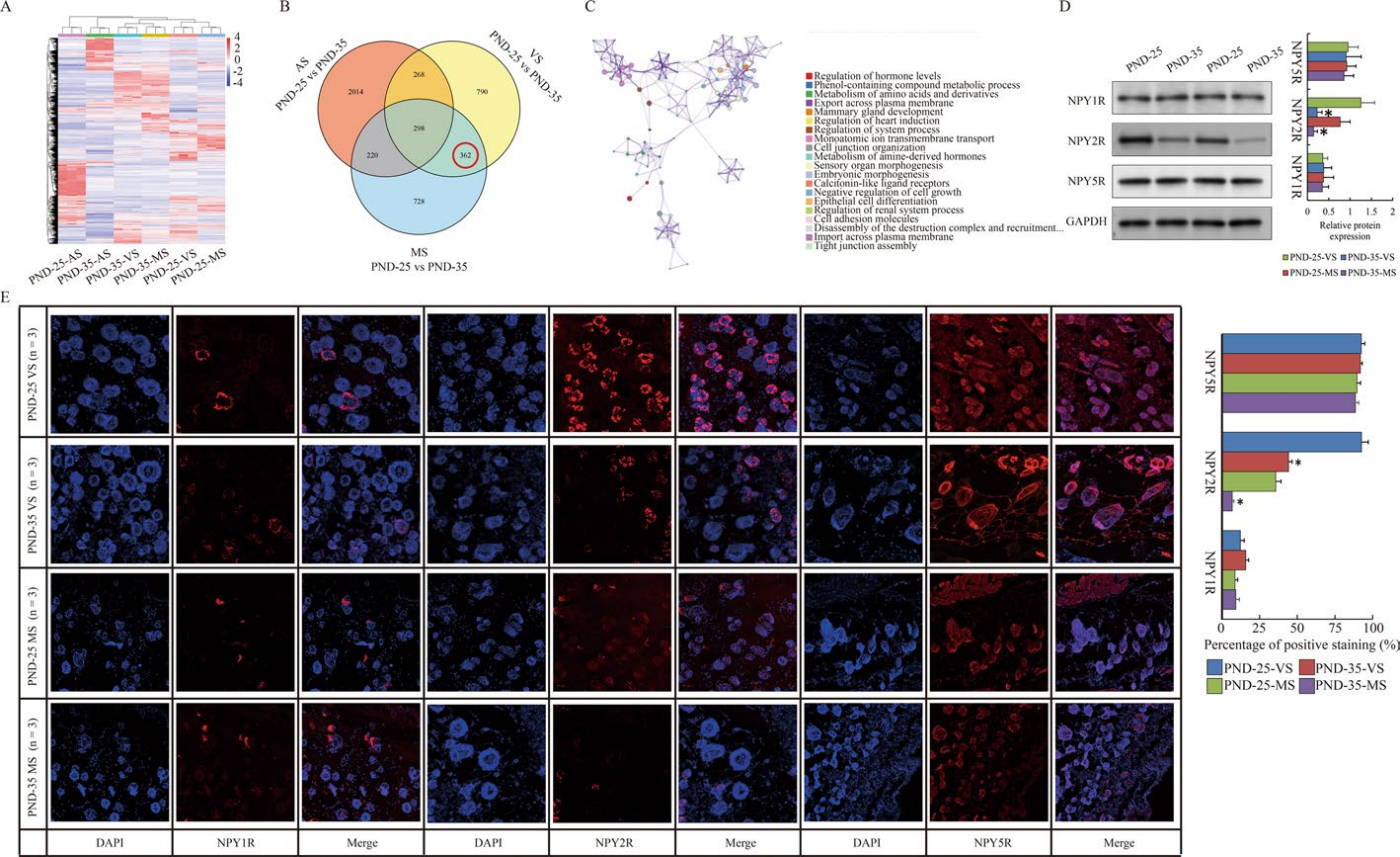
Down-regulated NPY2R in pubertal skin. **A** Heatmap shows the DEGs of MS, VS an AS compared between PND-25 and PND-35. **B** Venn diagram shows the interested DEGs special for SG compared between PND-25 and PND-35. **C** Metascape analysis shows the GO and KEGG terms involved in interested DEGs for SG. **D** Western blot show the expression of NPY receptors in VS and MS of PND-25 and PND-35. **E** IF show the expression of NPY receptors in VS and MS of PND-25 and PND-35 (magnification with ×200). The SG specimens from two individuals were used for western blot and RNA sequencing. Statistical analysis and abbreviated description can be referred to Fig. 1 legend

Next, we studied the transcriptome changes of these skin tissues by RNA sequencing. Differential expressed genes (DEGs) compared between PND-25 and PND-35 were obtained (Fig. 2A, Additional file 1: Table S1). Due to the primary concern on DEGs for SG, abdominal skin samples without SGs was excluded, and the remaining subset of 362 genes (127 up-regulated and 235 down-regulated in PND-25 *vs* PND-35) were considered as the putative special DEGs for SGs (Fig. 2B). “Metascape” showed that GO and KEGG analysis on these 362 DEGs showed that hormone regulation, gland development, cell junction and adhesion as well as epithelial differentiation were affected along with puberty onset (Fig. 2C). Here, we observed that *neuropeptide Y receptor Y2* (*Npy2r*) was significantly down-regulated in PND-35 (PND-25 *vs* PND-35: log_2_ FC = 1.62, *p* = 0.018 in volar skin; log_2_ FC = 1.73, *p* = 0.005 in mammary skin) (Additional file 1: Table S1), implying that the corresponding substrate NPY might exert as an important ligand for the function of SG. Consistently, only the protein expression of NPY2R in NPY receptor family (NPY1R, NPY2R and NPY5R) was reduced both in volar and mammary skin tissues of PND-35 compared to PND-25 (Fig. 2D, E). Interestingly, NPY2R antagonist SF-11 was subcutaneously administrated to PND-35 rat for 24 h, and resulted in the reduced expression of KRT5 and KRT10, as well as compromised the sebum level (Fig. 1B, C). All these data above suggested that NPY2R was supposed to be attributed to sebum synthesis.

### Heterodimer of NPY2R/NPY5R interacted with phosphorylated NFATc1

**Fig. 3:**
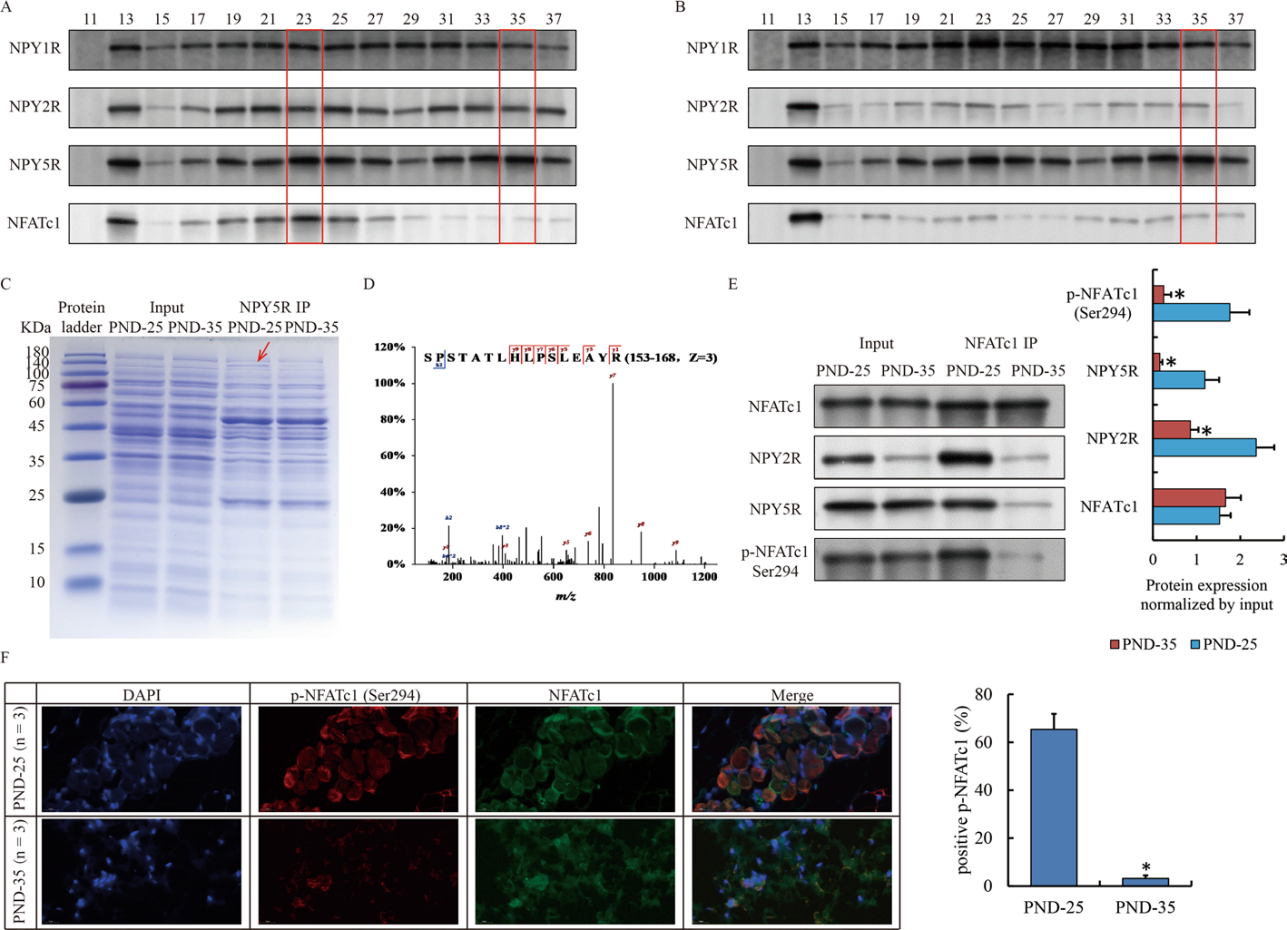
Special protein complex of NPY2R/NPY5R/NFATc1 in SG before puberty onset. **A**, **B** Gel filtration assay show the distribution of NPY receptors and NFATc1 in VS of PND-25 (**A**) and PND-35 (**B**). Red frames highlighted the fraction of potential target protein peak. **C** Coomassie brilliant blue staining shows the proteome interacted with NPY5R in VS of PND-25 and PND-35. Red arrow highlights a specific band at 140 kDa only appears in PND-25. **D** Original secondary mass spectrum shows the most confident protein (NFATc1) identified by mass spectrum. **E** Western blot confirm the interaction between NFATc1 and NPY2R/NPY5R via phosphorylated form. **F** IF show the localization of NFATc1 and p-NFATc1 at Ser294 in VS of PND-25 and PND-35 (magnification ×200). The SG specimens from two individuals were used for gel filtration, immunoprecipitation and western blot. The SG specimens from three individuals were used for immunofluorescence. Statistical analysis and abbreviated description can be referred to Fig. 1 legend

Due to the dimer form of NPY receptor on cytomembrane [23], we studied the potential heterodimer of NPY2R with NPY1R or NPY5R using gel filtration assay. Our findings indicated that NPY1R was located in different protein fraction from NPY2R, and was likely to form multiple complex with other unknown proteins in vola of PND-25 because only multiple peaks superimposed would show the sustained high expression in a series of continuous fraction (Fig. 3A). In turn, NPY2R and NPY5R clearly paired up around Fraction 23 and 35 (Fig. 3A). By contrast, due to the reduced expression of NPY2R in PND-35, NPY5R still appeared exactly in the same Fraction 35 as PND-25, but completely lost in Fraction 25, whereas the distribution of NPY1R was not affected (Fig. 3B). The discovery above suggested that one complex containing heterodimer of NPY2R/NPY5R was deteriorated in PND-35 owing to the reduction of NPY2R. To figure out this variable complex and the underlying mechanism behind, NPY5R was captured via immunoprecipitation by antibody. We noticed an interested band around 140 kDa, which obviously weakened in PND-35 compared to PND-25 (Fig. 3C), and identified it as nuclear factor of activated T cells 1 (*Nfatc1*) by mass spectrum (Fig. 3D). The interaction between NFATc1 and NPY2R/NPY5R was validated by gel filtration (Fig. 3A) and immunoprecipitation (Fig. 3E). In PND-35, we observed that NFATc1 was dispersed and the total protein was substantially reduced, suggesting that NFATc1 was more likely to exist in the free form (Fig. 3B). As previously reported, the function of NFATc1 was closely related to its phosphorylation [24, 25]. We further observed that p-NFATc1 at Ser294 interacting with NPY2R was abundant in PND-25 but almost deficient in PND-35 (Fig. 3A, B, E). Immunofluorescence also uncovered a remarkable reduction of phosphorylated NFATc1 in PND-35 (Fig. 3F). Taken together, we concluded that NFATc1 could substantially interact with NPY2R in sebocytes in PND-25.

### Genome-wide occupancy of NFATc1 enhanced transcription activities of sebocytes

**Fig. 4:**
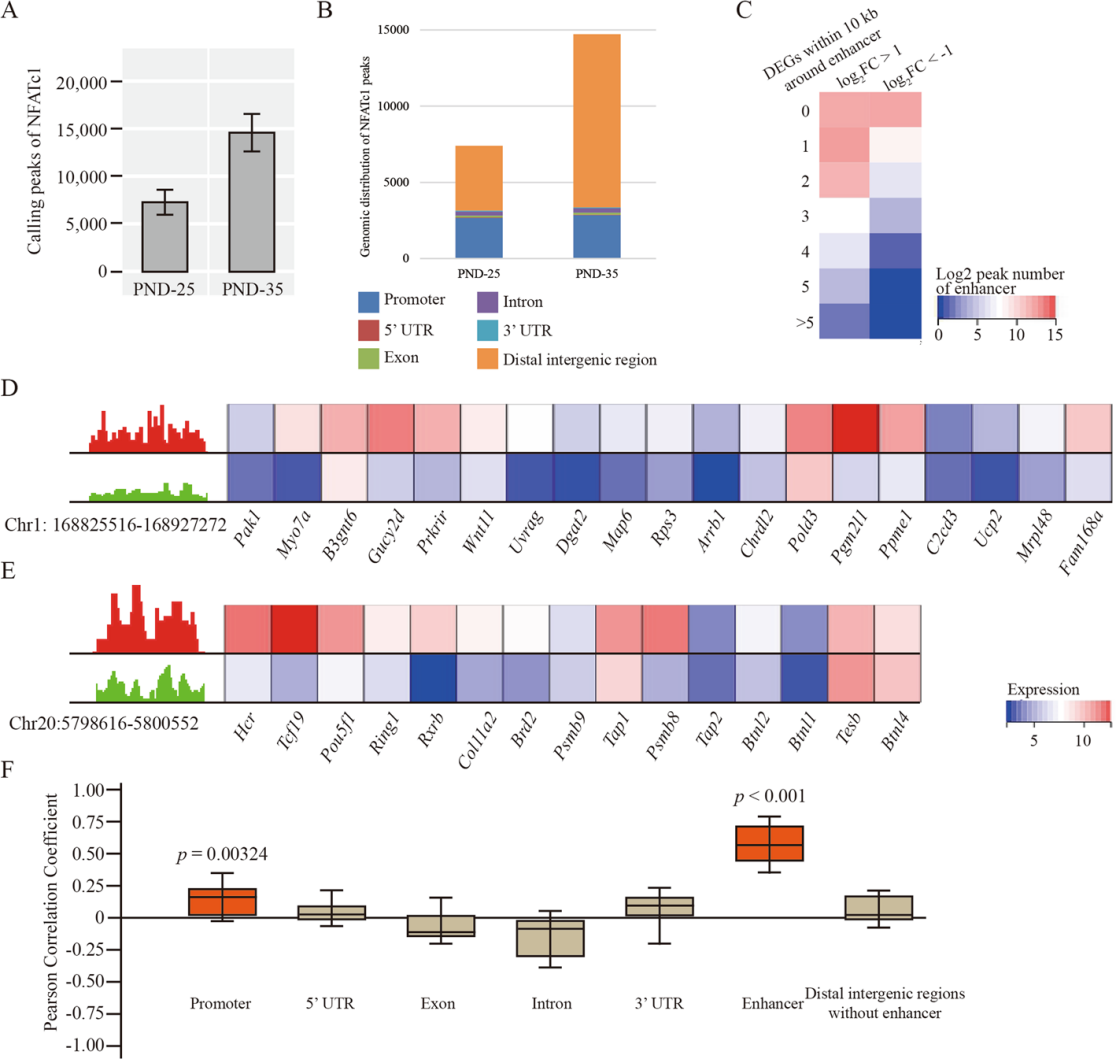
Genome-wide occupancy of NFATc1 at enhancers in VS. **A** Overall calling peaks of NFATc1 in VS of PND-25 and PND-35 with two biological repetitions. **B** The genomic distribution of NFATc1 peaks. **C** Heatmap shows the count of DEGs (left: up-regulated, right: down-regulated DEGs) within 10 kb around differentially NFATc1 binding enhancers compared between PND-25 and PND-35. **D**, **E** IGV and heatmap show the two examples of NFATc1 peak at Chr1: 168825516–168927272 (**D**) as well as Chr20:5798616–5800552 (**E**) and the followed the transcription of gene within 10 kb distance in PND-25 and PND-35. **F** Pearson correlation analysis shows the coefficient of correlation between differential NFATc1 binding ability at each regions and target gene transcription. Red boxes indicate the coefficient with *p*-value less than 0.05 while brown ones indicate *p*-value without statistical significance. The SG specimens from two individuals were used for ChIP sequencing

NFATc1 enters the nucleus and acts as a transcription factor after dephosphorylation. Therefore, we further studied the genome-wide occupancy of NFATc1 in sebocytes. Our observation indicated that the abundance of NFATc1 on genome was more robust in PND-35 than PND-25 (Fig. 4A). Moreover, we noticed that NFATc1 binding at the intergenic regions was increased in PND-35 compared PND-25 (Fig. 4B). Compared with known enhancer elements of rat species using EnhancerAtlas Database (http://www.enhanceratlas.org/) [26], it was notable that there were 154 enhancer regions overlapping with these significantly increased binding peaks of NFATc1 compared between PND-35 and PND-25 (log_2_FC > 1, *p* < 0.05). Simultaneously, 126 of these 154 enhancers contained at least three DEGs within 10 kb range (Fig. 4C). IGV showed two canonical enhancers (Chr1: 168825516–168927272, chr20: 5798616–5800552, Rnor5) followed by a series of genes involved in sebaceous gland synthesis *Ucp2* [27], and *Pou5f1* [28] (Fig. 4D, E). On the other hand, no significant correlation or weak correlation was found between differential gene expression and differential NFATc1 binding between PND-25 and PND-35 on other regions except enhancer (Fig. 4F). These data above suggested that the differentially binding of NFATc1 on enhancers could indeed govern the target genes’ transcription in the system of sebaceous glands.

### Inhibition of DYRK1A promoted the nuclear translocation and activity of NFATc1

**Fig. 5:**
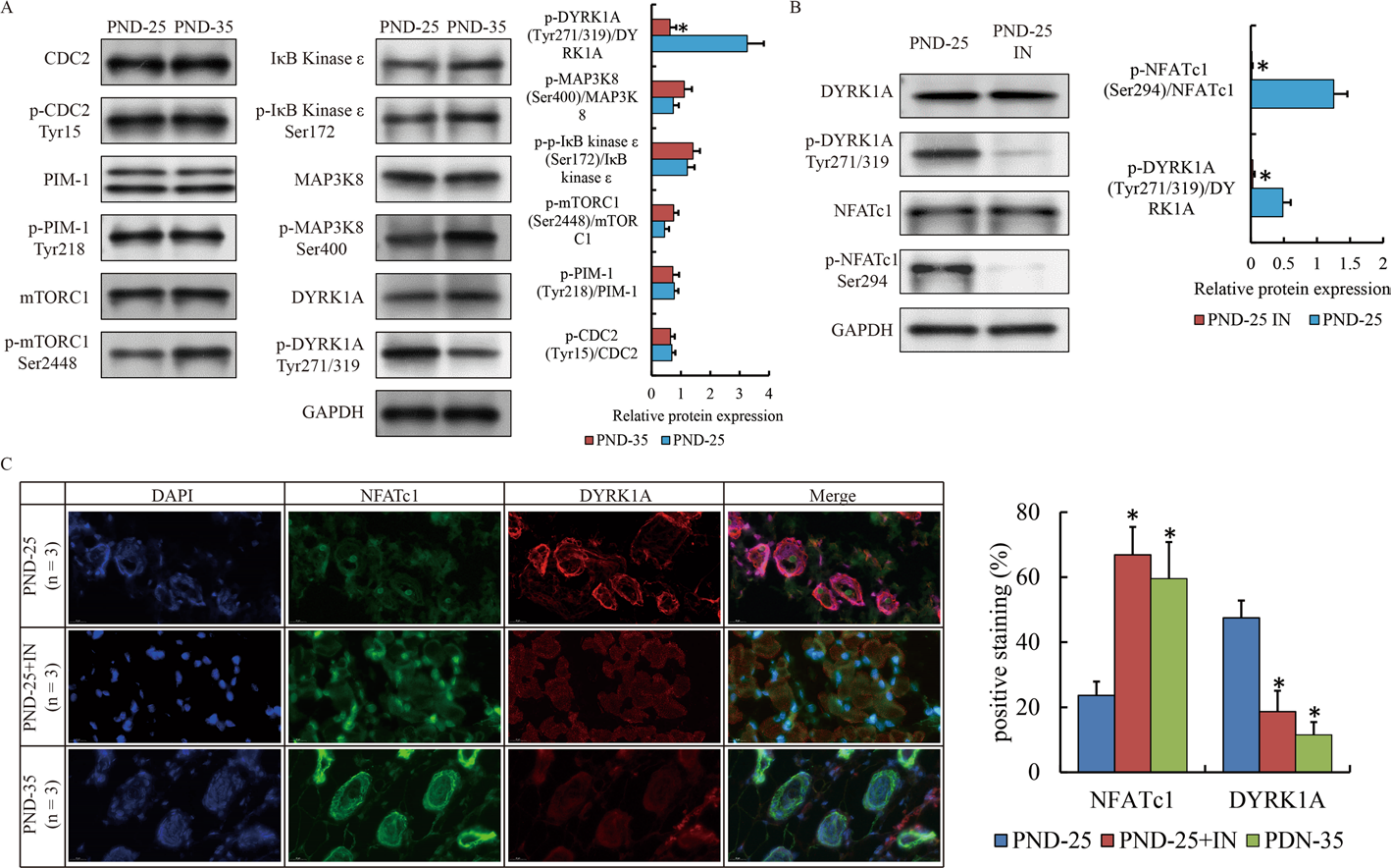
The activity of NFATc1 induced by DYRK1A in early puberty. **A** Western blot show the expression of NFATc1 related phosphokinases and their phosphorylation in VS of PND-25 and PND-35. **B** Western blot show the phosphorylation of NFATc1 affected by DYRK1A inhibitor. **C** IF show the localization of NFATc1 and phosphorylated NFATc1 in VS of PND-25, PND25 with DYRK1A inhibitor and PND-35 (magnification ×200). The SG specimens from two individuals were used for western blot. The SG specimens from three individuals were used for immunofluorescence

Next, we focused on which phosphatase or phosphokinase was attributed to NFATc1 dephosphorylation in sebaceous glands of PND-35. Kinases such as CDC2 [29], PIM-1 [24], mTORC1 [30] and IκB Kinase ε [31], MAP3K8 [32] as well as DYRK1A [33, 34] have been reported to affect the phosphorylation and nuclear translocation of NFATc1 in other tissues or diseases. Expression of these kinases were detected by western blot. We observed that all these proteins appeared to be equal between PND-25 and PND-35 (Fig. 5A). Strikingly, the phosphorylation of DYRK1A (Tyr271/319) was significantly reduced, while mTOR (Ser2448) was slightly elevated in PND-35 compared to PND-25 (Fig. 5A). The activation of mTOR apparently had the opposite effect on nuclear translocation of NFATc1. Therefore, we only examined the effect of DYRK1A using DYRK1A inhibitor to the subcutaneous vola of PND-25, and observed that the phosphorylation of NFATc1 was also compromised (Fig. 5B). Meanwhile, the staining of NFATc1 was abundant in nucleus accompanied with reduced localization of DYRK1A in PND-25 induced by DYRK1A inhibitor or PND-35 (Fig. 5C). Taken together, our findings suggested that DYRK1A was responsible for the nuclear translocation and activity of NFATc1 in sebocytes.

## Discussion

Relevant epidemiological studies have figured out that hormone, inflammation, allergies, stress, irritation, cosmetics, blood lipids, dairy food intake, smoking, hyperpigmentation, ethnicity, and genetics are implicated in acne [35]. Nevertheless, hormone imbalances may render acne unresponsive to conventional therapy, suggesting that endocrine regulation plays a vital role in acnegenesis. Moderate-to-severe acne affects around 20% of young people and severity correlates with pubertal maturity [36]. Exception of the androgen-stimulated sebum production, other hormones, including estrogens, growth hormone, insulin, insulin-like growth factor-1, glucocorticoids, adrenocorticotropic hormone, and melanocortins all participate in the pathophysiology of acne [37]. Corresponding to them, sebum cells express a variety of receptors, including peptide hormone receptors, neurotransmitter receptors, and steroid and thyroid hormone receptors. Various hormones and mediators acting through sebum cell receptors finally determine the choreography of acne pathologies.

NPY has been shown to be the most widely distributed neuropeptide throughout multiple tissues in a variety of organisms, and has pleiotropic roles throughout the body in the central biological processes [38]. NPY is elevated in the affected skin and/or circulation of humans suffering from various skin pathologies, including atopic dermatitis, cutaneous melanoma, psoriasis, and vitiligo [39]. The biological behavior of NPY exerts through the interaction with five, G protein-coupled, membrane-bound, NPY Y receptors (Y1R, Y2R, Y4R, Y5R) [40]. However, the molecular role of NPY and receptors in sebaceous regulation and acne is still poorly understood to date. NPY4R is not expressed in all skin samples by RNA sequencing, therefore we majorly focus on the other three receptors in current study.

Now our observations provide novel molecular evidence of NPY and NPY2R in SGs. RNA sequencing has indicated that the transcript of *Npy2r* is reduced in volar and mammary tissue with abundant SGs after pubertal initiation (Additional file 1: Table S1). Furthermore, NPY2R inhibition and compensation can indeed affect the growth of sebaceous progenitors and sebum production (Fig. 1B, C). NPY signaling occurrs via the NPY2 receptor (NPY2R), stimulating PI3K, MAPK, mTORC1 and NFAT activation [41]. NPY-NPY2R signaling is benefit to vasodilation, and cell proliferation, but not good for cell differentiation and migration [42–44]. Therefore, the functional activation of SGs as well as the reduced *Npy2r* after puberty onset are logically self-consistent.

Moreover, the biological significance of the differential expression of *Npy2r* lies in the NPYR heterodimer. Different combinations of NPYR heterodimer showing the tissue and disease specificity are usually subject to the subcellular localization of NPYR [45], NPY concentrations and target proteins coordinated with the receptor-ligand interactions [46]. Our findings suggest that dimerization of NPY2R and NPY5R is specific in system of SG, and loss of NPY2R substantially deteriorate the interaction with NPY5R. Beyond that, we have unexpectedly discovered that NPY5R appear to participate in two kinds of complex, the larger of which independent of NPY2R (Fraction 35) is likely a homodimer, and is worthy of further investigation into its function on NPY signal conduction.

Furthermore, subsequent observations of IP and mass spectrum indicate that NFATc1 is a time-specific regulator interacting with NPY2R/NPY5R. NFATc1 is calcium–calcineurin dependent regulator that contributes to osteoclastogenesis [47], cardiac valves formation [48], and regulatory T-cells suppression [49]. In classical osteoclastogenesis activated by RANKL, PLCγ binds to the IP3 receptor (IP3R), induces calcium release from the endoplasmic reticulum, and then increases calcium levels to activate calcineurin, which is followed by the dephosphorylation and nuclear translocation of NFATc1 [50]. Although the issue about calcium–calcineurin is not covered in this paper, we still believe that NFATc1 is dependent on their regulation whatever it is inhibited in SGs in the early puberty or activated after puberty onset. The relationship between calcium and the action of DYRK1A or other phosphokinases is worthy of further exploration in future studies. Moreover, as a master transcription factor, NFATc1 can bind to many enhancers for remote transcriptional regulation of terrestrial sebaceous gland related genes in our system. But this does not mean that NFATc1 has similar genome-wide occupancy in other disease states and different tissue types. For example, we compared the genomic binding data of NFATc1 in one article on pancreatic cancer [51], and the results were completely different (Data now shown). We have reason to believe that NFATc1 has a tissue-specific regulatory mode in the nucleus, showing more unknown epigenetic regulatory factors interacting with it, which is a new research direction developed from NPY2R.

## Conclusions

In summary, we figure out a novel molecular mechanism of NPY2R/NFATc1/DYRK1A regulatory axis in sebum synthesis. Our findings establish a new regulatory relationship in the field of sebaceous gland secretion, and have a certain theoretical value for the advancement of the research on skin diseases, skin repair, medical cosmetology, pharmaceuticals and cosmetics.

## Supplementary Information

**Additional file 1: Table S1.** Gene information of DEGs compared between PND-25 and PND-35. DEGs are defined as log2 fold change > 1 or < − 1, and *p* < 0.05. MS: mammary skin; VS: volar skin; AS: abdominal skin.

## Data Availability

The datasets supporting the conclusions of this article are included within the article and its additional file.

## Abbreviations

SG: Sebaceous gland
HPT: Hypothalamic-pituitary-thyroid
HPA: Hypothalamic-pituitary-adrenocortical
HPG: Hypothalamic-pituitary-gonad
NPY: Neuropeptide Y
NPYR: Neuropeptide Y receptor
PND: Post-natal day
DEG: Differential expressed gene
KRT5/10: Keratin 5/10
NFATc1: Nuclear factor of activated T cells 1
CDC2: Cell division cycle 2
PIM-1: Pim-1 proto-oncogene, serine/threonine kinase
mTORC1: Mammalian target of rapamycin C1
DYRK1A: Dual specificity tyrosine phosphorylation regulated kinase 1A
IGV: Integrative genomics viewer
